# Prognostic Value of Decreased High-Density Lipoprotein Cholesterol Levels in Infective Endocarditis

**DOI:** 10.3390/jcm11040957

**Published:** 2022-02-12

**Authors:** Rosa Zampino, Fabian Patauner, Arta Karruli, Domenico Iossa, Maria Paola Ursi, Lorenzo Bertolino, Anna Maria Peluso, Fabiana D’Amico, Giusi Cavezza, Emanuele Durante-Mangoni

**Affiliations:** 1 Department of Advanced Medical and Surgical Sciences, University of Campania “Luigi Vanvitelli”, Piazza Luigi Miraglia, 2, 80138 Napoli, Italy; rosa.zampino@unicampania.it (R.Z.); fabian@patauner.it (F.P.); mariapaola.ursi@hotmail.com (M.P.U.); lorenzo.bertolino91@gmail.com (L.B.); annapelu11@gmail.com (A.M.P.); 2 Unit of Infectious & Transplant Medicine, AORN Ospedali dei Colli—Ospedale Monaldi, Piazzale Ettore Ruggieri, 80131 Napoli, Italy; domenico.iossa@unicampania.it (D.I.); fabianadamico@libero.it (F.D.); 3 Department of Precision Medicine, University of Campania “Luigi Vanvitelli”, Via de Crecchio, 7, 80138 Napoli, Italy; arta.karruli@unicampania.it (A.K.); giusicavezza@gmail.com (G.C.)

**Keywords:** infective endocarditis, HDL, prognosis, mortality, hypolipidemia

## Abstract

(1) Background: Simple parameters to be used as early predictors of prognosis in infective endocarditis (IE) are lacking. The aim of this study was to evaluate the prognostic role of high-density-lipoprotein cholesterol (HDL-C) and also of total-cholesterol (TC), low-density-lipoprotein cholesterol (LDL-C), and triglycerides, in relation to clinical features and mortality, in IE. (2) Methods: Retrospective analysis of observational data from 127 consecutive patients with a definite diagnosis of IE between 2016 and 2019. Clinical, laboratory and echocardiography data, mortality, and co-morbidities were analyzed in relation to HDL-C and lipid profile. (3) Results: Lower HDL-C levels (*p* = 0.035) were independently associated with in-hospital mortality. HDL-C levels were also significantly lower in IE patients with embolic events (*p* = 0.036). Based on ROC curve analysis, a cut-off value was identified for HDL-C equal to 24.5 mg/dL for in-hospital mortality. HDL-C values below this cut-off were associated with higher triglyceride counts (*p* = 0.008), higher prevalence of *S. aureus* etiology (*p* = 0.046) and a higher in-hospital mortality rate (*p* = 0.004). Kaplan–Meier survival analysis showed higher 90-day mortality in patients with HDL-C ≤ 24.5 mg/dL (*p* = 0.001). (4) Conclusions: Low HDL-C levels could be used as an easy and low-cost marker of severity in IE, particularly to predict complications, in-hospital and 90-day mortality.

## 1. Introduction

Infective endocarditis (IE) is a microbial disease affecting the endocardium, showing a high morbidity and mortality, ranging from 12% in native valve endocarditis (NVIE) to nearly 25% in prosthetic valve endocarditis (PVIE), and with a 1-year mortality >30% [1,2,3]. Heart failure, sepsis, and embolism are common complications of IE [2,4,5,6,7] with heart failure being the most common cause of mortality [8,9]. Severely ill IE patients may commonly show a profound derangement of their physiology, influencing several metabolic and immuno-inflammatory domains, including lipid metabolism.

Indeed, the effects of acute infectious processes on lipid profiles have been the focus of several investigations [10,11,12]. Lipid profiles are generally characterized by reductions in total cholesterol (TC), high-density lipoprotein (HDL-C), and low-density lipoprotein cholesterol (LDL-C) levels and, often, by an increase in triglycerides [10]. This pattern has been observed in various infectious diseases and appears strongly linked to the severity of illness; it appears to be highest in sepsis with septic shock, where the inflammatory response to infection is more pronounced [10,13]. A condition of hypocholesterolemia, including reduction of TC, LDL-C and HDL-C, has been associated with a worse prognosis in patients with infection or sepsis [14,15,16]. Moreover, this form of hypolipidemia appeared to be superior compared to procalcitonin and C-reactive protein in predicting mortality in sepsis [17,18].

The possible role and significance of hypolipidemia in IE have not yet been established. In a study on 54 left-sided IE cases, Kahveci et al. found that HDL-C levels were lower in patients with IE compared with healthy controls and that among the former with a complicated disease course, HDL-C levels were lowest [19]. Whether evaluation of HDL-C levels per se could improve the prognostic assessment in patients with acute IE remains at present unclear.

Accordingly, the aim of the present study was to evaluate the prognostic significance of serum lipid levels in IE.

## 2. Patients and Methods

### 2.1. Study Design

This was a retrospective analysis of clinical and biochemical data obtained in the context of a prospective observational study ongoing at our center. Included were patients admitted to the Unit of Infectious and Transplant Medicine, AORN Ospedali dei Colli-Ospedale Monaldi, Naples, Italy, between January 2016 and July 2019, for suspected IE and for whom the ESC diagnostic criteria (ESC 2015 guidelines) for definite IE were met. In January 2016 our clinical research protocol included determination of the serum lipid profile in suspected IE cases on the day of admission. Only patients with serum lipid levels available were included in this analysis.

### 2.2. Analysed Variables

Collected patient data included: age, sex, clinical presentation, anthropometric parameters, co-morbidities, embolic events. Haemato-chemical data analyzed were: complete blood count, C-reactive protein (CRP), erythrocyte sedimentation rate (ESR), creatinine, estimated glomerular filtration rate (eGFR, calculated according to the Modified Diet in Renal Disease formula), glycemia, total/HDL/LDL cholesterol, triglycerides. For the purpose of this study, chronic kidney disease (CKD) was defined as an eGFR <60 mL/min/1.73 m^2^ BSA documented to be present before IE onset. ESRD was defined as an admission eGFR <15 mL/min. All haemato-chemical parameters were obtained on admission by routine biochemical methods used in our hospital central laboratory. Triglycerides, TC and LDL-C were measured by a colorimetric assay, HDL-C by a catalase elimination method.

A trans-thoracic echocardiogram (TTE) was performed in all patients within 72 h of admission, followed by a transesophageal echocardiogram (TEE) where needed. Details about IE characteristics (on native, prosthetic, or cardiac implantable electronic devices), endocardial vegetations (size and location), and microbial etiology were also collected. Finally, data on surgical procedures and mortality were recorded.

This study was approved by our University Ethics Committee (prot. AOC/0011110/2020) and was compliant with the 1975 Declaration of Helsinki and its later amendments. Each patient gave his/her informed consent to participate in the study and consented to data collection at the time of IE diagnosis.

### 2.3. Statistical Analysis

Statistical analysis was performed on data obtained at the time of hospital admission.

Continuous or numerical variables are presented as the mean ± standard deviation (SD) or median and interquartile range (IQR), while categorical variables were presented as numbers and percentages. The statistical significance of the observed differences was evaluated by the chi-square test or Fisher’s exact test for categorical variables and by the Mann–Whitney *U* test or Kruskal–Wallis test for numerical variables. Correlation between numerical variables was analyzed by Spearman’s coefficient. Variables associated with the outcome of interest at univariate analysis (*p* < 0.05) were subsequently included in multivariate logistic regression models in order to identify covariates independently associated with the same outcome. A receiver operating characteristic (ROC) curve analysis was performed regarding HDL-C values to establish a cut-off of this parameter best discriminating the risk of death in included IE patients. On the basis of this cut-off, the study population was subsequently divided into two subgroups. Survival analysis was carried out by the Kaplan–Meier method.

All the analyses were performed with the aid of the SPSS 25 software (IBM, Armonk, NY, USA) and R software (CRAN^®^ 3.3.4), with the assumption of a *p*-value ≤ 0.05 as indicative of statistical significance of the observed differences on two-sided tests.

## 3. Results

### 3.1. Study Population

During the study period, 166 IE patients were admitted to our center. Of them, 127 (76.5%) had lipid profiles tested on admission and were enrolled in this study. The median age was 65 years (IQR: 56–72 years) with a higher prevalence of males (67.7%). Fifty-nine patients presented infection on a native valve, 32 on a prosthetic valve, and 27 on a cardiac implantable electronic device. The general characteristics of the study group are described in Table 1.

The most common etiologies of IE were *coagulase-negative Staphylococci* in 23.7% of cases and *Staphylococcus aureus* and *Streptococcus* spp. in 19.6% of cases each (Table 1), whilst the most prevalent comorbidities observed were ischemic heart disease (25.1%), chronic heart failure (22.8%) and diabetes mellitus (22%).

### 3.2. HDL and Lipid Profile in IE Patients

**Figure 1:**
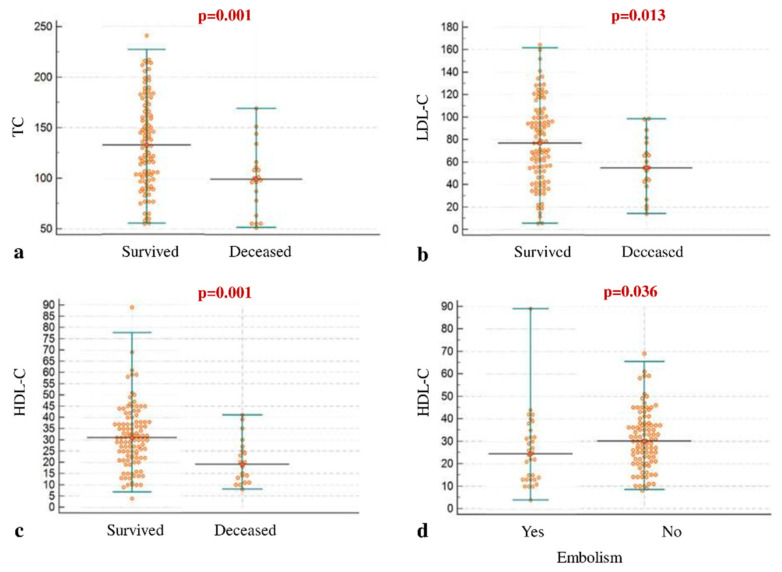
Dot-plot graphs depicting the distribution of total cholesterol (**a**), LDL-C (**b**), and HDL-C (**c**) values in surviving and deceased patients and HDL-C distribution in patients with and without embolic events (**d**). HDL-C, high-density lipoprotein-cholesterol; LDL-C, low-density lipoprotein-cholesterol; TC, total cholesterol.

**Figure 2:**
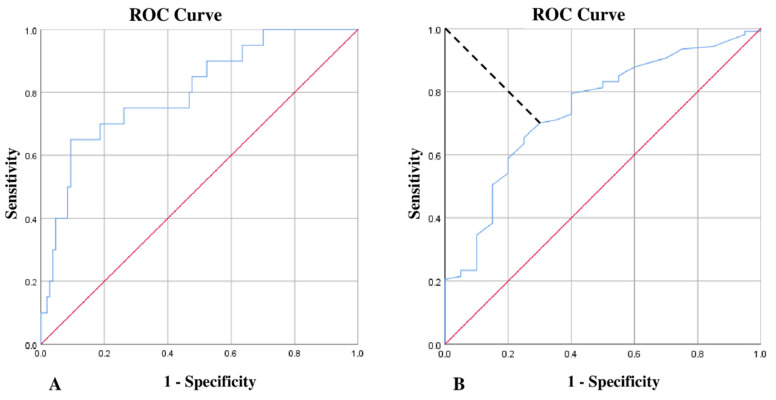
Panel (**A**): ROC curve analysis of the logistic regression model of HDL-C association with in-hospital mortality, adjusted for age and comorbidities (see Table 2). Area under the ROC curve: 0.804, 95% CI 0.698–0.910, *p* < 0.001). Panel (**B**): ROC curve analysis of HDL-C best cut-off for hospital mortality in IE. Area under the ROC curve: 0.743, 95% CI 0.628–0.857; *p* = 0.001).

Median levels of TC were 123 mg/dL (IQR 97–162), of HDL-C 29 mg/dL (IQR 20–37), and of LDL-C 69.6 mg/dL (IQR 45.2–96), whilst median triglyceride levels were 118 mg/dL (IQR 92–156.7) (Table 1). A condition of hypolipidemia, defined as total cholesterol less than 120 mg/dL and/or LDL-C less than 50 mg/dL, was observed in 60 patients (47.2%). There was a significant inverse correlation between HDL-C and triglycerides (r = −0.31; *p* < 0.001). For the univariate analysis, baseline lipid levels were significantly lower in deceased IE patients than survivors. Specifically, both TC (99 vs. 133 mg/dL, *p* = 0.001), HDL-C (19 vs. 31 mg/dL, *p* = 0.001) and LDL-C (54.7 vs. 76.6 mg/dL, *p* = 0.013) were lower in IE patients who did not survive. Diabetes mellitus and chronic kidney disease were also higher in deceased patients and eGFR at admission was lower among those who died during admission. The other variables analyzed, including most of the known factors affecting IE prognosis, did not show a statistically significant association with in-hospital mortality (Table 2, Figure 1). In particular, in this specific cohort of IE patients, no statistically significant association with in-hospital mortality was observed for *S. aureus* etiology, cardiac surgery performance, age, and type of IE (native, prosthetic, cardiac implantable electronic device) (Table 2). No other etiology appeared related to IE outcome (Supplementary Table S1), although only 1 patient with Streptococcal IE died in hospital. For the multivariate analysis, an independent association with mortality was found for HDL-C (odds ratio 0.937, 95% CI 0.882–0.996, *p* = 0.037) and for chronic kidney disease (odds ratio 5.171, 95% CI 1.197–22.340, *p* = 0.028). For an increase of 1 mg/dL of HDL-C, the risk of mortality was reduced by 6.3%, whereas an increase of 10 mg/dL reduced it by 48%. HDL-C association with in-hospital mortality holds true after controlling for age and comorbidities (Figure 2A).

HDL-C levels were also significantly lower in IE patients who experienced septic embolic events (median 24 vs. 30 mg/dL in those without embolism; *p* = 0.036; Supplementary Table S2, Figure 1). Prediction of IE complicated by embolism was further increased by combining evaluation of higher CRP levels and lower HDL-C levels (Supplementary Table S2).

### 3.3. HDL Cholesterol Cut-Off

Based on prior studies focusing on HDL-C [19], we calculated the area under the ROC curve for HDL-C, with hospital mortality as the outcome variable. In this ROC curve analysis, it was possible to identify a cut-off value for HDL-C equal to 24.5 mg/dL showing a specificity of 65% and a sensitivity of 71% for hospital mortality (area under the receiver operating characteristic curve: 0.743, 95% CI 0.628–0.857; *p* = 0.001) (Figure 2B).

Dividing the 127 patients into 2 groups based on the identified cut-off (respectively, *n* = 46 and *n* = 81), we observed (Table 3) that patients with HDL-C values below this cut-off presented higher CRP levels (median 11 vs. 6.5 mg/dL, *p* = 0.004), higher neutrophil counts (*p* = 0.004), a lower TC (100.5 vs. 145 mg/dL, *p* < 0.001) and LDL-C level (55 vs. 86 mg/dL, *p* < 0.001), and higher triglycerides (142 vs. 108 mg/dL, *p* < 0.001). In addition, these patients showed a significantly higher in-hospital mortality rate (30.4% vs. 7.4%; *p* = 0.002). An association of HDL-C levels with IE etiology was also observed (Table 3). In particular, there was a difference in the prevalence of pathogens between the two groups (*p* = 0.026), specifically a higher prevalence of *S. aureus* infection in subjects with HDL-C below the cut-off (35.9% vs. 15.2%). In contrast, a lower prevalence of *Streptococcus* spp. etiology was evident in subjects with HDL-C below the cut-off (7.7% vs. 30.6%). The single patient with IE due to *Streptococcus* spp. who died during hospitalization had indeed an HDL-C value as low as 14 mg/dL. Supplementary Figure S1 details the median values of HDL-C across subgroups of different microbial etiology. Among comorbidities, an association with lower HDL-C levels was also found for chronic kidney disease. A trend was observed for an association between lower HDL-C and larger vegetations as well as a higher rate of embolic complications (Table 3).

For the multivariable analysis, in-hospital mortality emerged as the strongest independent correlate of HDL-C values lower than the 24.5 mg/dL cut-off (odds ratio 9.2, 95%CI 2.0–41.9) (Table 3). In addition, a higher neutrophil count, a higher triglyceride level as well as IE etiology were independently associated with a level of HDL-C under the cut-off (Table 3).

**Figure 3:**
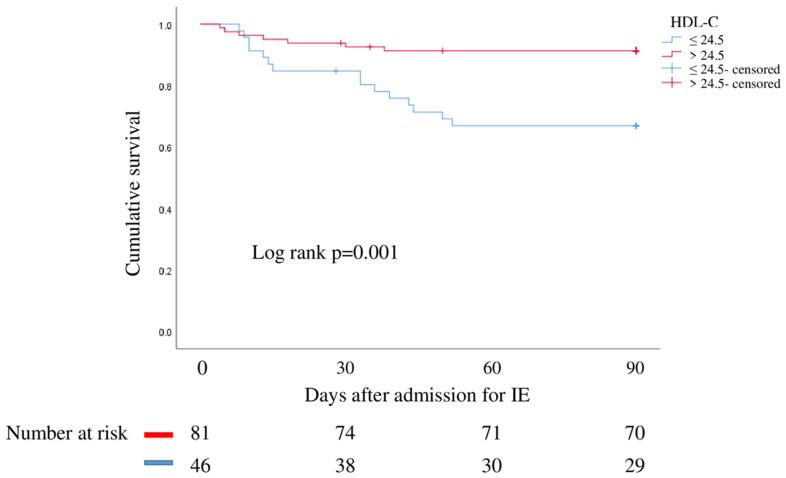
Kaplan–Meier survival analysis at 90 days after hospital admission for IE. Red line indicates patients with HDL-C ≤ 24.5 mg/dL. Blue line denotes patients with HDL-C > 24.5 mg/dL.

Finally, survival analysis was carried out by the Kaplan–Meier method. This analysis confirmed that mortality at 90 days after admission for IE in subjects with HDL-C levels below the cut-off was significantly higher (log-rank *p* = 0.001) (Figure 3).

## 4. Discussion

The results of the present study suggest that lower levels of TC, LDL-C, and, most importantly, HDL-C at hospital admission for IE are associated with a significantly higher in-hospital and 90-day mortality. In IE patients, HDL-C was an independent predictor of in-hospital mortality. Of note, HDL-C levels were not affected by comorbidities such as diabetes mellitus and CKD, in contrast to prior studies in settings other than IE [20,21]. In addition, HDL-C levels were significantly inversely related with triglycerides and such a pro-atherogenic/dysmetabolic lipid pattern was strongly associated with a worse prognosis, even in the short term. Lower HDL-C also tended to associate with larger vegetations and a higher rate of embolic complications. Overall, our findings suggest that HDL-C can be a very easy to obtain and inexpensive biomarker of prognosis in patients with IE.

Several mechanisms could be in place which could explain the observed prognostic value of HDL-C in IE. In severe bacterial infections, HDL-C levels may be reduced due to either decreased production (decreased apoA1 production [12] and decreased lecithin–cholesterol acyltransferase activity [22,23]) or increased clearance (via raised serum amyloid A [24,25], increased serum phospholipase A2 and endothelial cell lipase activity, or enhanced triglyceride incorporation through cholesteryl ester transfer protein activity [26,27]). The higher is the severity of infection, the larger the HDL lowering effect. Our observation of a correlation between low HDL-C and high CRP and neutrophil count supports this paradigm.

A relationship exists between HDL particles and the immune-inflammatory system. An indirect effect of HDLs on the control of excessive inflammation induced by infection has been suggested [10]. In the specific setting of IE, HDL particles might influence several pathways: (i) detoxification of lipoteichoic acid, expressed by Gram-positive cocci, preventing its binding to toll-like receptors 2 and 6 [28]; (ii) prevention of macrophage activation, by induction of key transcriptional repressors of innate immune response genes [29]; (iii) attenuation of endothelial cell activation [12,30,31]; and (iv) inhibition of platelet aggregation [10,12,31]. All of these mechanisms may well associate with a more complicated course of IE. However, the question remains as to whether low HDL-C levels are the cause or a consequence of a more severe infection/inflammation in IE.

In IE patients with lower HDL-C levels, there was a higher propensity towards embolic complications. This finding could be explained by the interaction between HDL particles, platelets, and the coagulation system [31]. HDL-C may reduce platelet activity through direct binding to scavenger receptors expressed on their surface as well as decreased generation of diacylglycerol and activation of protein kinase C [32]. HDL-C may also play a direct role in the coagulation cascade, stimulating protein C activation and its ability to inactivate Factors V/VIII, and reducing procoagulant effects of platelet anionic phospholipids [33]. In addition, HDL-C is inversely related to the levels of plasminogen activator inhibitor-1 and may therefore promote plasmin generation and fibrinolysis [34]. All or some of these effects might explain the observed association of low HDL-C levels with larger vegetation size and higher embolic risk in IE [32,34,35]. The clinical use of HDL-C levels together with other markers of embolic events in IE, including CRP [36,37], appears to increase the predictive value of both parameters in the diagnosis of embolic complications of IE, as suggested by our data (Supplementary Table S2).

Further studies are surely needed to better understand the pathophysiological implications of hypolipidemia, chiefly low HDL-C, in IE.

In this study, it was interesting to note that admission CRP, the most common marker of inflammation, as well as neutrophil count, did not show a prognostic value, at variance with prior investigations [38,39], but were significantly associated with lower HDL-C levels. This finding could suggest HDL-C levels exert an independent effect on the severity of inflammation occurring in response to IE.

Concerning IE etiology, *S. aureus* was more prevalent in patients with lower HDL-C levels, while the opposite was observed for *Streptococcus* spp. infections. This observation further supports the hypothesis of an association between HDL-C levels and severity of IE, notoriously greater with the former and smaller with the latter etiology [2].

Our data need to be assessed in light of recent studies on the potential effects of lipid-lowering agents in inflammatory and infectious conditions. A positive effect of the addition of fibrates and/or statins was shown in experimental models and in human studies in sepsis [40,41,42]. The same consideration may be carried out for recombinant HDLs that showed beneficial effects on both coagulation and inflammatory response in experimental models of sepsis [43,44]. To the best of our knowledge, no similar data have been generated in either experimental or clinical models of IE. Unfortunately, we did not collect data on the use of statins or fibrates before IE onset in our patients. An additional weakness of this study was the relatively low number of subjects analyzed, which could have contributed to the absence of statistically significant associations between common IE mortality risk factors [45,46,47,48] and outcome in our cohort. Moreover, our study was limited by the inclusion of only patients for whom lipid profile at hospital admission was available, a condition that could have partially influenced some of our results.

## 5. Conclusions

In conclusion, low HDL-C levels could be used as an easily available and low-cost marker of severity in IE, helping physicians to identify patients who are at risk for developing complications and death during hospitalization and in the 90 days after admission. Further studies are surely warranted to obtain mechanistic explanations for our findings.

## Figures and Tables

**Table 1:** Characteristics of the study cohort (*n* = 127).

Parameter	Result	Normal Ranges
Age (years), median [IQR]	65 [56–72]	
Sex, *n* (%)		
*Male*	86 (67.7)	
*Female*	41 (32.3)	
Fever, (°C), median [IQR]	39 [38–39]	
CRP, (mg/dL), median [IQR]	8.1 [3.6–14.1]	<1.0
ESR, (mm/h), median [IQR]	56 [36.7–78.7]	2–14
White blood cells, (cells/µL), median [IQR]	10,550 [7960–15,500]	4500–11,000
Neutrophils, (cells/µL), median [IQR]	9250 [5600–12,700]	2900–7000
Creatinine, (mg/dL), median [IQR]	1.0 [0.8–1.4]	0.6–1.1
eGFR MDRD, (mL/min), median [IQR]	70 [50–99]	≥90
Glycemia, (mg/dL), median [IQR]	109 [91–137]	74–106
Cholesterol, (mg/dL), median [IQR]		
*Total*	123 [97–162]	<200
*HDL*	29 [20–37]	40–60
*LDL*	69.6 [45.2–96]	50–129
Triglycerides, (mg/dL), median [IQR]	118 [92–156.7]	50–150
Etiology *n* (%)		
*S. aureus*	25 (19.6)	
*Enterococcus* spp.	22 (17.3)	
*Streptococcus* spp.	25 (19.6)	
*Coagulase-negative staphylococci*	30 (23.7)	
*Other pathogens*	9 (7.2)	
*Negative blood cultures*	16 (12.6)	
Type of IE, *n* (%)		
*Native valve*	59 (46.5)	
*Prosthetic valve*	32 (25.2)	
*CIED*	27 (21.2)	
Other *	9 (7.1)	
Comorbidities		
Ischemic heart disease, *n* (%)	32 (25.1)	
Chronic heart failure, *n* (%)	29 (22.8)	
Diabetes mellitus, *n* (%)	28 (22)	
Chronic kidney disease, *n* (%)	16 (11.9)	
Intravenous drug use, *n* (%)	4 (3.1)	
Cardiac surgery, *n* (%)	79 (62.2)	
Embolism, *n* (%) **	32 (22.5)	
*Spleen, n (%)*	18 (14.1)	
*Lung, n (%)*	12 (9.4)	
*Kidney, n (%)*	6 (4.7)	
*Brain, n (%)*	5 (3.9)	
*Vascular periphery, n (%)*	2 (1.5)	
*Liver, n (%)*	1 (0.7)	
*Other, n (%)*	2 (1.5)	
In-hospital mortality, *n* (%)	20 (15.7)	

Data are expressed as number and percentage or median and interquartile range (IQR). IQR, interquartile range; CRP, C-reactive protein; ESR, erythrocyte sedimentation rate; eGFR, estimated glomerular filtration rate; MDRD, modified diet in renal disease; HDL, high-density lipoprotein; LDL, low-density lipoprotein; CIED, cardiac implantable electronic devices. * Include IE on mitral valve repair (valvuloplasty) (*n* = 4), Contegra valvulated pulmonary tube (*n* = 3), Melody percutaneous pulmonary valve (*n* = 1), and transcatheter aortic valve implant (*n* = 1). ** multiple sites of embolism were present in some patients.

**Table 2:** Univariable and multivariable analysis of factors possibly associated with IE outcome.

	Outcome of Hospitalization Univariable Analysis			Multivariable Analysis	
Parameter	Deceased (*n* = 20)	Discharged Alive (*n* = 107)	*p*-Value	O.R. [95% CI]	*p*
Age (year), median [IQR]	68 [62–75]	65 [55–72]	0.197	1.020 [0.979–1.061]	0.347
Sex			0.604		
Male	15 (75)	71 (66.3)			
Female	5 (25)	36 (33.7)			
Temp max	39 [38.2–39.2]	39 [38–39]	0.448		
Type of IE, *n* (%)			0.684		
*Native*	10 (50)	49 (45.8)			
*Prosthetic*	6 (30)	26 (24.3)			
*CIED*	4 (20)	23 (21.5)			
*Other*	0 (0)	9 (8.4)			
*S. aureus* etiology	4 (20)	21 (19.62)	0.449		
Vegetation size *, *n* (%)			0.265		
>10 mm	11 (55)	53 (49.5)			
≤10 mm	8 (40)	20 (18.6)			
Cardiac surgery, *n* (%)	16 (80)	63 (58.8)	0.134		
Ischemic heart disease, *n* (%)	5 (25)	27 (25.2)	1		
Chronic heart failure, *n* (%)	5 (25)	24 (22.4)	0.777		
Diabetes mellitus, *n* (%)	8 (40)	20 (18.6)	**0.044**	2.188 (0.662–7.233)	0.199
Chronic kidney disease, *n* (%)	8 (40)	8 (7.4)	**0.001**	**5.171 (1.197–22.340)**	**0.028**
Embolism	5 (25)	27 (25.2)	1		
Cholesterol, (mg/dL), median [IQR]					
*Total*	99 [66.7–114.7]	133 [102–169]	**0.001**		
*HDL*	19 [11.5–26.5]	31 [22–38]	**0.001**	0.937 (0.882–0.996)	**0.037**
*LDL*	54.7 [39.4–74.7]	76.6 [51.2–99.6]	**0.013**	0.993 (0.974–1.012)	0.474
Triglycerides, (mg/dL), median [IQR]	109 [88–152]	121 [93–164]	0.281		
White blood cells, (cells/µL), median [IQR]	14,880 [7897–20,045]	10,400 [7960–14,400]	0.162		
Neutrophils, (cells/µL), median [IQR]	12,725 [6517–18,192.5]	8200 [5460–12,260]	0.101		
CRP, (mg/dL), median [IQR]	11 [3.2–20]	7.8 [3.7–13]	0.313		
Creatinine, (mg/dL), median [IQR]	1.4 [0.73–1.72]	0.96 [0.8–1.3]	0.114		
eGFR MDRD, (mL/min), median [IQR]	45.5 [30.7–85.2]	74 [55–100]	**0.042**	1.006 (0.992–1.020)	0.423
Glycemia, (mg/dL), median [IQR]	118.5 [96.7–167.6]	109 [91–136]	0.429		
ESR, (mm/h), median [IQR]	50 [32–77]	56 [37–81]	0.978		

Data are expressed as number and percentage or median and interquartile (IQR) * Vegetation size was precisely recorded in 92 patients. CIED, cardiac implantable electronic devices; CRP, C-reactive protein; HDL, high-density lipoprotein; IQR, interquartile range; LDL, low-density lipoprotein.

**Table 3:** Univariable and multivariable analysis of factors associated with a lower HDL cholesterol level.

	Univariable Analysis			Multivariable Analysis	
Parameter	HDL		*p*	O.R. [95% CI]	*p*
	≤24.5 (*n* = 46)	>24.5 (*n* = 81)			
Age (years), median [IQR]	64.5 [44–72]	68 [57–72]	0.343		
Sex, *n* (%)			0.844		
*M*	32 (69.5)	54 (66.6)			
*F*	14 (30.4)	27 (33.3)			
Fever, (°C), median [IQR]	39 [38–39.5]	39 [38–39]	0.347		
CRP, (mg/dL), median [IQR]	11 [4.7–18.1]	6.5 [2.3–11.5]	**0.004**	0.986 [0.944–1.031]	0.543
ESR, (mm/h), median [IQR]	60 [34–77]	54 [37–81]	0.709		
Neutrophils, (cells/µL), median [IQR]	11,295 [6152–16,700]	7590 [5145–10,820]	**0.004**	1.000 [1.000–1.000]	0.221
Creatinine, (mg/dL), median [IQR]	1.2 [0.7–2.1]	0.9 [0.8–1.2]	0.062		
eGFR MDRD, (mL/min), median [IQR]	60.5 [33–123.2]	74 [61–95.5]	0.156		
Glycemia, (mg/dL), median [IQR]	109 [86–168]	110 [91.5–135]	0.49		
Cholesterol, (mg/dL), median [IQR]					
*Total*	100.5 [76.5–122.7]	145 [115.5–179.5]	**0**		
*LDL*	55 [35.4–69.8]	86 [58.9–103.6]	**0**		
Triglycerides, (mg/dL), median [IQR]	142 [113.5–198]	108 [88.5–141]	**0**	**1.011 [1.003–1.019]**	**0.008**
Left sided IE, *n* (%)	27 (58.7)	64 (79.0)	**0.08**		
Right sided IE, *n* (%)	16 (34.8)	15 (18.5)			
Etiology, *n* (%)			**0.026**	**0.664 [0.444–0.993]**	**0.046**
*S. aureus*	14 (35.9)	11 (15.2)			
*Enterococcus* spp.	9 (23.1)	13 (18.1)			
*Streptococcus* spp.	3 (7.7)	22 (30.6)			
*CoNS*	10 (25.6)	20 (27.7)			
*Other pathogens*	3 (7.7)	6 (8.4)			
Diabetes mellitus, *n* (%)	11 (23.9)	17 (20.9)	0.824		
Chronic heart failure, *n* (%)	9 (19.5)	20 (24.6)	0.661		
Chronic kidney disease, *n* (%)	10 (21.7)	6 (7.4)	**0.026**	4.442 [0.885–22.292]	0.07
Ischemic heart disease, *n* (%)	13 (28.2)	19 (23.4)	0.674		
Cardiac surgery, *n* (%)	27 (58.6)	52 (64.1)	0.33		
Vegetation size, (mm), median [IQR]	18 [10–20]	13 [10–19]	0.106		
Embolism, *n* (%)	16 (34.7)	16 (19.7)	0.088		
In-hospital mortality, *n* (%)	14 (30.4)	6 (7.4)	**0.002**	**9.292 [2.059–41.934]**	**0.004**

Data are expressed as number and percentage or median and interquartile range (IQR). CoNS, coagulase-negative *Staphylococci*; CRP, C-reactive protein; eGFR, estimated glomerular filtration rate; ESR, erythrocyte sedimentation rate; HDL, high-density lipoprotein; IQR, interquartile range; LDL, low-density lipoprotein; MDRD, modification of diet in renal disease.

## Data Availability

The dataset used for this article is available on request from the corresponding author.

## Supplementary Materials

The following are available online at https://www.mdpi.com/article/10.3390/jcm11040957/s1, Figure S1: HDL-Cholesterol levels at admission in patient with *Staphylococcus aureus*, *Enterococcus* spp., *Streptococcus* spp., Coagulase-negative *Staphylococci*, other infective endocarditis pathogens and culture negative IE patients; in blue patients discharged alive, in red patients died during admission; the dashed line represents the HDL-C cut-off of 24.5 mg/dL. CoNS, Coagulase-negative *Staphylococci*; HDL-C, high-density lipoprotein cholesterol; IE, Infective Endocarditis. Table S1: In-hospital mortality according with different etiological agent of IE and in particular specific *Streptococcus* species. Table S2: Lipid levels according to performance of cardiac surgery and occurrence of embolic events.

## Appendix A

Members of the Monaldi Hospital Cardiovascular Infection Study Group:

Emanuele Durante-Mangoni, Domenico Iossa, Lorenzo Bertolino, Maria Paola Ursi, Fabiana D’Amico, Arta Karruli, Mohammad Ramadan, Roberto Andini, Rosa Zampino (Internal Medicine); Mariano Bernardo, Giuseppe Ruocco (Microbiology); Giovanni Dialetto, Franco Enrico Covino, Sabrina Manduca (Echocardiography); Alessandro Della Corte Marisa De Feo (Cardiac Surgery); Stefano De Vivo (Electrophysiology); Maria Luisa De Rimini (Nuclear Medicine); Nicola Galdieri (Intensive Care Unit).
