# Supplementary material for: Prognostic Value of Decreased High-Density Lipoprotein Cholesterol Levels in Infective Endocarditis
Source: J Clin Med. 2022 Feb 12;11(4):957. doi: 10.3390/jcm11040957 (PMC8877683; doi:10.3390/jcm11040957)
Supplement: Supplementary file 1 [file jcm-11-00957-s001.zip › jcm-1560223-supplementary.pdf]

Etiology	Discharged alive	Deceased	p value
Staphylococcus aureus	21	4	0.449
Coagulase-negative Staphylococci	24	6	
Streptococcus spp	24	1	
Enterococcus spp.	20	2	
Other	8	1	
Streptococcus gallolyticus	13	1	
Streptococcus mitis	1	0	
Streptococcus gordonii	3	0	
Streptococcus salivarius	1	0	
Streptococcus viridans	1	0	
Streptococcus sanguinis	1	0	
Streptococcus agalactiae	2	0	
Streptococcus mutans	2	0	

Supplementary Table S1. In-hospital mortality according with different etiological agent of IE and in particular specific *Streptococcus* species.

	Univariate Analysis		
	Cardiac surgery		
**Parameter**	**Yes (n=79)**	**No (n=43)**	**P**
Cholesterol, (mg/dl), median [IQR]			
Total	132 [92 – 169]	117 [103 – 151]	0.832
HDL	29 [20 – 38]	26 [19 – 37]	0.554
LDL	70.2 [41.2 – 98.2]	68.8 [55.6 – 90.2]	0.556
Triglycerides, (mg/dl), median [IQR]	118.5 [95.7 – 188]	121 [91 – 142]	0.312
	**Embolism**		
**Parameter**	**Yes (n=32)**	**No (n=95)**	**P**
Cholesterol, (mg/dl), median [IQR]			
Total	140.5 [99.2 – 163.7]	121 [97 – 162]	0.683
HDL	24.5 [13 – 34.2]	30 [21 – 38]	0.036
LDL	82.2 [52 – 99.1]	68.6 [42.4 – 95.3]	0.495
Triglycerides, (mg/dl), median [IQR]	135 [106 – 167.2]	116 [89 – 156.7]	0.151
CRP, (mg/dl), median [IQR]	11.5 [7.3 – 17.8]	6.35 [2.8 – 12.8]	0.040
HDL-C <24.5 mg/dl + CRP >median, N (%)	14 (43.7%)	16 (17%)	0.008

Data are expressed as number and percentage or median and interquartile range (IQR)

IQR, interquartile range; HDL, high density lipoprotein; LDL, low density lipoprotein; CRP, C-reactive protein

Supplementary Table S2. Lipid levels according to performance of cardiac surgery and occurrence of embolic events

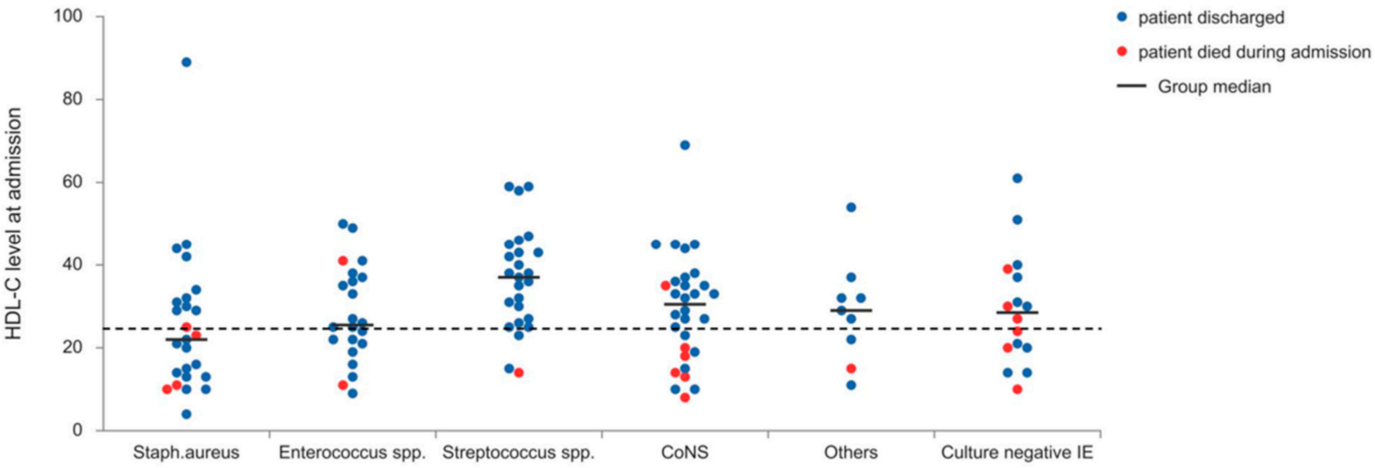

Supplementary figure S1. HDL-Cholesterol levels at admission in patient with *Staph aureus*, *Enterococcus* spp., *Streptococcus* spp., Coagulase negative *Staphylococci*, other infective endocarditis pathogens and culture negative IE patients; in blue patients discharged alive, in red patients died during admission; the dashed line represents the HDL-C cut-off of 24.5 mg/dl. *CoNS, Coagulase-Negative Staphylococci; HDL-C, High Density Lipoprotein-Cholesterol; IE, infective endocarditis.*
